# Young Onset Neurocognitive Impairment, Amyloid Positivity, and Psychiatric Symptoms in Underrepresented Populations in the HABS‐HD Cohort

**DOI:** 10.1002/alz.094962

**Published:** 2025-01-09

**Authors:** Julia E. Culhane, Alyssa Arentoft, Vanessa A. Guzman, Heining Cham, Desiree Byrd, Amanda T. Calcetas, Monica Rivera Mindt

**Affiliations:** ^1^ Fordham University, New York, NY USA; ^2^ California State University Northridge, Northridge, CA USA; ^3^ Icahn School of Medicine at Mount Sinai, New York, NY USA; ^4^ CUNY, Queens College, Queens, NY USA

## Abstract

**Background:**

Adults from underrepresented populations (URPs), including non‐Latinx NLB (NLB) and Latinx adults, have higher Alzheimer’s disease and related dementia (ADRD) rates than non‐Latinx Whites (NLWs). Young onset dementia is diagnosed when symptom onset occurs before age 65, and little is known about mild cognitive impairment (MCI) and dementia rates in younger URPs. We examined neurocognitive diagnoses, amyloid positivity, and psychiatric symptoms in adults from URPs under 65.

**Methods:**

A total of 1,562 participants under 65 (*Mage* = 57.8, *SD* = 4.18; *Meducation* = 13.08, *SD* = 4.09; Ethnocultural Status: 28.9% NLB, 46.2% Latinx, 24.9% NLW; Gender: 65.5% Female) from the Health and Aging Brain Study‐Health Disparities (HABS‐HD) completed a neuropsychological (NP) battery and received Clinical Dementia Rating Scale (CDR) scores. Study partners completed the Physician’s Estimate of Duration for AD‐related psychiatric symptoms (e.g., mood changes, anxiety/nervousness). Diagnoses (i.e., cognitively unimpaired (CU), MCI, dementia) were conferred using HABS‐HD consensus diagnosis criteria. A subset of 756 participants (*M* = 57.4, *SD* = 4.21; 57.7% NLB, 27.6% Latinx) underwent PET imaging with Florbetaben (FBB); amyloid positivity was defined by an SUVR of 1.08. Chi‐square tests were computed to examine associations across ethnocultural groups (i.e., NLB, Latinx, and NLW) and neurocognitive diagnoses, amyloid positivity, and psychiatric symptoms.

**Results:**

The NLB group had significantly higher rates of MCI and lower rates of CU based on consensus diagnoses (*n* = 451; MCI = 30.6%, CU = 63.2%), compared to NLW (*n* = 389; MCI = 14.1%; CU = 80.7%, *X^2^* = 41.20, *p*<.001) and Latinx groups (*n* = 722; MCI = 18.6%; CU = 76.2%, *X^2^* = 40.09, *p*<.001). The NLB group also had higher rates of psychiatric symptoms, including mood changes (*X^2^* = 17.70, *p*<.001), anxiety/nervousness (*X^2^* = 13.05, *p* = .001), and aggression/irritability (*X^2^* = 15.29, *p*<.001) compared to NLW and Latinx groups. In contrast, amyloid positivity and dementia rates did not significantly differ across groups (all *ps*>.05).

**Conclusion:**

Young NLB participants in HABS‐HD had increased rates of MCI and psychiatric symptoms compared to NLW and Latinx participants. Also notable, there were no significant ethnocultural differences in amyloid positivity or dementia diagnosis. These patterns differ from those previously documented in primarily NLW samples, suggesting that psychosocial and possibly sociocultural factors may differentially affect cognitive health outcomes among middle‐aged NLB adults. Future research, with deeper sociocultural phenotyping, may help elucidate the potential mechanisms driving these differences.

